# Human Physiology

**Published:** 1875-11

**Authors:** 


### Bibliographical.

---

HUMAN PHYSIOLOGY FOR STUDENTS AND PRACTITIONERS OF MEDICINE. By John C. Dalton, M.D., Professor of Physiology and Hygiene in the College of Physicians and Surgeons, New York. Published by Henry C. Lea, Philadelphia. Cloth; pp. 825.

This, the sixth edition, contains eight hundred and twenty-five pages, with three hundred and sixteen beautiful and instructive illustrations. It is divided into three general sections. The first, devoted to nutrition; second, to the nervous system, and third, to reproduction. These sections are subdivided in chapters. The first contains seventeen chapters, treating respectfully of proximate principles, inorganic proximate principles, hydro-carbonaceous proximate principles, albuminous matters, coloring matters, crystallizable nitrogenous matters, food, digestion, absorption, the bile, production of glygogen and glucose in the liver, the blood, respiration, animal heat, the circulation, the lymphatic system, and the urine

Section second contains eight chapters. First, structure and function of nervous system, nervous irritability and mode of action, general arrangement of nervous system, the spinal cord, the brain, the cranial nerves, the sympathetic system, and the senses.

Section third contains eighteen chapters. First, the nature of reproduction and origin of plants and animals, sexual generation and mode of accomplishment, the egg and the female organs of generation, the seminal fluid and the male organs of generation, periodical ovulation and the function of menstruation, the corpus luteum and its connection with menstruation and pregnancy, development of impregnated egg, formation of embryo in fowl's egg, development of accessory organs in impregnated egg, umbilical vesicle, amnion and allantois, development of impregnated egg in membranes in human species, development of decidual membrane and attachment to uterus, the placenta, discharge of fœtus from placenta and regeneration of uterine tissues, development of nervous system, organs of sense, skeleton, limb, development of alimentary canal and appendages, development of kidneys and internal organs of generation, development of vascular system, development of body after birth.

It will be useless to attempt a correct analysis of the contents of this valuable work. The high position occupied in scientific circles by the author, and by the wide spread popularity of this treatise on physiology, which has made it a standard work, and the text book in many of the leading schools of the country, bespeak for it the patronage to which it is justly entitled.

